# Genotype-degree of hemolysis correlation in hereditary spherocytosis

**DOI:** 10.1186/s12864-023-09364-8

**Published:** 2023-06-06

**Authors:** Yimeng Shi, Yuan Li, Xiawan Yang, Xiaoxia Li, Guangxin Peng, Xin Zhao, Xu Liu, Yufei Zhao, Jing Hu, Xiangrong Hu, Baohang Zhang, Kang Zhou, Yang Yang, Youzhen Xiong, Jianping Li, Huihui Fan, Wenrui Yang, Lei Ye, Liping Jing, Li Zhang, Fengkui Zhang

**Affiliations:** Anemia Therapeutic Centre, State Key Laboratory of Experimental Hematology, National Clinical Research Center for Blood Diseases, Haihe Laboratory of Cell Ecosystem, Institute of Hematology & Blood Diseases Hospital, Chinese Academy of Medical Sciences & Peking Union Medical College, Tianjin, 300020 China

**Keywords:** Hereditary spherocytosis, Red blood cell lifespan, Degree of hemolysis, Levitt’s carbon monoxide breath test, Next-generation sequencing

## Abstract

**Background:**

Hereditary spherocytosis (HS) is a common inherited hemolytic anemia, caused by mutations in five genes that encode erythrocyte membrane skeleton proteins. The red blood cell (RBC) lifespan could directly reflect the degree of hemolysis. In the present cohort of 23 patients with HS, we performed next-generation sequencing (NGS) and Levitt’s carbon monoxide (CO) breath test to investigate the potential genotype-degree of hemolysis correlation.

**Results:**

In the present cohort, we identified 8 ANK1,9 SPTB,5 SLC4A1 and 1 SPTA1 mutations in 23 patients with HS, and the median RBC lifespan was 14(8–48) days. The median RBC lifespan of patients with ANK1, SPTB and SLC4A1 mutations was 13 (8–23), 13 (8–48) and 14 (12–39) days, respectively, with no statistically significant difference (*P* = 0.618). The median RBC lifespan of patients with missense, splice and nonsense/insertion/deletion mutations was 16.5 (8–48), 14 (11–40) and 13 (8–20) days, respectively, with no significant difference (*P* = 0.514). Similarly, we found no significant difference in the RBC lifespan of patients with mutations located in the spectrin-binding domain and the nonspectrin-binding domain [14 (8–18) vs. 12.5 (8–48) days, *P* = 0.959]. In terms of the composition of mutated genes, 25% of patients with mild hemolysis carried ANK1 or SPTA1 mutations, while 75% of patients with mild hemolysis carried SPTB or SLC4A1 mutations. In contrast, 46.7% of patients with severe hemolysis had ANK1 or SPTA1 mutations and 53.3% of patients with severe hemolysis had SPTB or SLC4A1 mutations. However, there was no statistically significant difference in the distribution of mutated genes between the two groups (*P* = 0.400).

**Conclusion:**

The present study is the first to investigate the potential association between genotype and degree of hemolysis in HS. The present findings indicated that there is no significant correlation between genotype and degree of hemolysis in HS.

## Background

Clinically, hereditary spherocytosis (HS) is characterized by peripheral blood spherocytosis and nonimmune chronic extravascular hemolysis. The pathology of HS is based on mutations in five genes that encode erythrocyte membrane skeleton proteins, namely, ANK1, SPTB, SPTA1, SLC4A1 and EPB42, resulting in erythrocyte membrane loss and erythrocyte spheroidization. Spherocytes are more rigid and less capable of passing through the splenic sinusoids, where they are trapped and subsequently phagocytosed by splenic macrophages [1].

The association between genotype and phenotype in HS has long been debated as different membrane skeleton protein gene abnormalities involve different types and degrees of membrane skeleton protein deletion. Previous clinical studies have reported conflicting findings. Some studies suggested that SPTA1 mutations are associated with more severe anemia and frequent transfusion requirements but that, patients with SLC4A1 deficiencies have a milder phenotype with higher hemoglobin (Hb) levels and lower reticulocyte counts [2, 3]. However, other studies have reported that mutations in ANK1 and SPTB are associated with a more severe phenotype as indicated by lower Hb levels and higher reticulocyte counts [4]. The phenotypic difference between ANK1 and SPTB mutations is not significant. It has also been reported that there is more severe anemia and frequent splenectomy in ANK1 mutations compared to SPTB mutations [5, 6]. However, previous studies have mainly focused on the correlation between genotype and the severity of anemia, which does not accurately reflect the effect of genotype on clinical phenotype due to its dependence on the degree of hemolysis and bone marrow erythropoiesis compensative capacity. Hemolytic parameters, such as indirect bilirubin (IBIL), haptoglobin and lactate dehydrogenase, do not accurately reflect the degree of hemolysis due to their susceptibility to various circumstances. Thus, the effect of genotype on the degree of hemolysis alone is rarely reported due to methodological limitations of hemolysis measurement. To investigate this relationship, we analysed the red blood cell (RBC) lifespan in 23 HS patients with different mutations as RBC lifespan may directly reflect the degree of hemolysis [7].

## Results

### Clinical features of HS patients

The clinical characteristics and median laboratory parameters of the 23 patients are shown in Table 1. The median age at diagnosis was 30 (7–73) years old, and 10/24 (41.7%) of the patients were male. The median values of RBC lifespan, Hb level, mean corpuscular volume (MCV), mean corpuscular hemoglobin concentration (MCHC), absolute reticulocyte count (ARC), percentage of reticulocyte, erythropoietin (EPO) level and IBIL were 14(8–48) days, 106(87–154) g/L, 91.3(78.2-103.4) fl., 347(317–387) g/L, 397.7(181.8-661.6) ×10^9^/L, 12.14(3.78–23.24)%, 41.25(14.40-132.52) mIU/ml and 72.1(21.8–236.0) µmol/L, respectively.

**Table 1:** Patient characteristics according to the degree of hemolysis classification in HS

Clinical characteristics	Total(n = 23)	Mild(n = 8)	Severe(n = 15)
Male, n (%)	10/24(41.7%)	3(37.5%)	7(46.7%)
Age(years), median(range)	30(7–73) n = 23	27(9–45) n = 8	30(7–73) n = 15
RBC lifespan(days), median(range)	14(8–48) n = 23	21.5(15–48) n = 8	12(8–14) n = 15
Hb(g/L), median(range)	106(87–154) n = 23	109(100–154) n = 8	101(87–118) n = 15
MCV (fl.), median(range)	91.3(78.2-103.4) n = 22	90.3(78.2–97.3) n = 8	92.9(80.9-103.4) n = 14
MCHC(g/L), median(range)	347(317–387) n = 22	359(317–387) n = 8	346(328–373) n = 14
ARC(×10^9^/L),median(range)	397.7(181.8-661.6) n = 23	355.5(181.8-521.8) n = 8	471.1(213.2-661.6) n = 15
Ret (%), median(range)	12.14(3.78–23.24) n = 23	11.76(3.78–15.86) n = 8	12.63(7.78–23.24) n = 15
EPO (mIU/ml), median(range)	41.25(14.40-132.52) n = 8	27.21(17.71–36.71) n = 2	47.36(14.40-132.52) n = 6
IBIL (µmol/L), median(range)	72.1(21.8–236.0) n = 12	46.5(21.8–92.6) n = 4	84.2(40.3–236.0) n = 8
Osmotic fragility test(positive), n (%)	22/22(100%)	8/8(100%)	14/14(100%)
EMA (%), median(range)	29.10(18.73–44.96) n = 19	36.51(26.79–44.94) n = 8	28.96(18.73–44.96) n = 11

The degree of hemolysis was classified as mild(RBC lifespan>14 days) or severe(RBC lifespan ≤ 14 days).Abbreviations: Hb, hemoglobin; MCV, mean corpuscular volume; MCHC, mean corpuscular hemoglobin concentration; ARC, absolute reticulocyte count; Ret, reticulocyte; EPO, erythropoietin; IBIL, indirect bilirubin

### Mutation spectrum of HS patients

In the present study, 24 mutations were identified in 23 HS patients, and the detailed mutation data of the patients are shown in Table 2. In the present cohort, 4 membrane protein genes with pathogenic mutations were detected. In total, 8/23 (34.8%) patients had ANK1 mutations, 9/23 (39.1%) patients had SPTB mutations, 5/23 (21.7%) patients had SLC4A1mutations and 1/23 (4.3%) patients had SPTA1 mutations. Moreover, no EPB42 mutation was detected in the present study. In addition, 4/23 patients presented with VUS, and the mutations were considered to be pathogenic according to the clinical diagnosis and family history.

**Table 2:** Pathogenic mutations in the HS cohort

Patient ID	Inheritance pattern	Gene	Location	cDNA change	Protein change	Pathogenicity	Zygosity	RBC lifespan(days)
1	unknown	ANK1	Exon2	c.106dupG	p.Q36Yfs*5	5-P	heterozygous	11
2	De novo	ANK1	Exon31-Exon38 Del	Exon31-Exon38 Del	NA	4-LP	heterozygous	14
3	De novo	ANK1	exon29	c.3376_3377del	p.A1126Hfs*95	4-LP	heterozygous	18
4	De novo	ANK1	intron16	c.1801-17G > A	splicing	5-P	heterozygous	11
5	De novo	ANK1	exon2	c.28-1G > T	splicing	5-P	heterozygous	23
6	unknown	ANK1	exon29	c.3239-1G > C	splicing	5-P	heterozygous	14
7	unknown	ANK1	exon29	c.3301 C > A	p.P1101T	3-VUS	heterozygous	8
8	unknown	ANK1	exon6	c.427-2 A > G	splicing	4-LP	heterozygous	12
9	De novo	SPTB	exon14	c.2291_2295delGGCTGinsAGCCGGTGG	p.R764Qfs	5-P	heterozygous	10
10	AD	SPTB	exon28	c.6001 C > T	p.R2001C	3-VUS	heterozygous	48
11	De novo	SPTB	exon7	c.850delG	p.A284Qfs*20	5-P	heterozygous	15
12	unknown	SPTB	exon11	c.1628G > A	p.W543X	4-LP	heterozygous	8
13	De novo	SPTB	exon13	c.1816dupC	p.Q606Pfs*15	5-P	heterozygous	20
14	unknown	SPTB	exon10	c.1080dupG	p.N361Efs*30	4-LP	heterozygous	13
15	De novo	SPTB	exon19	c.4266 + 1_4266 + 2del GCinsAT	splicing	5-P	heterozygous	40
16	De novo	SPTB	exon13	c.1738 C > T	p.Q580X	5-P	heterozygous	11
17	unknown	SPTB	exon8	c.826_833del	p.Tyr276GlnfsTer17	4-LP	heterozygous	13
18	unknown	SLC4A1	exon17	c.2281 A > T	p.I761F	3-VUS	heterozygous	12
19	AD	SLC4A1	exon18	c.2423G > A	p.R808H	4-LP	heterozygous	13
20	unknown	SLC4A1	exon17	c.2278 C > T	p.R760W	5-P	heterozygous	20
21	unknown	SLC4A1	exon9	c.808G > T	p.G270X	4-LP	heterozygous	14
22	AD	SLC4A1	exon19	c.2510 C > T	p.T837M	4-LP	heterozygous	39
23	AR	SPTA1	exon3	c.382delG	p.E128Kfs*6	4-LP	compound heterozygous	14
	AR	SPTA1	exon51	c.7022 C > T	p.A2341V	3-VUS		

Mutation nomenclature is based on the following NCBI Reference sequence transcript numbers: ANK1: NM_001142446.2; SPTB: NM_001024858.3; SLC4A1: NM_000342.3; SPTA1: NM_003126.4; and EPB42: NM_000119.3. Sequence variants were interpreted following recommendations from the American College of Medical Genetics and Genomics [8], and identified variants were described using standard terminology:1-B: benign, 2-LB: likely benign, 3-VUS: variant of unknown significance, 4-LP: likely pathogenic, and 5-P: pathogenic

### RBC lifespan in HS with different mutated genes

**Fig. 1:**
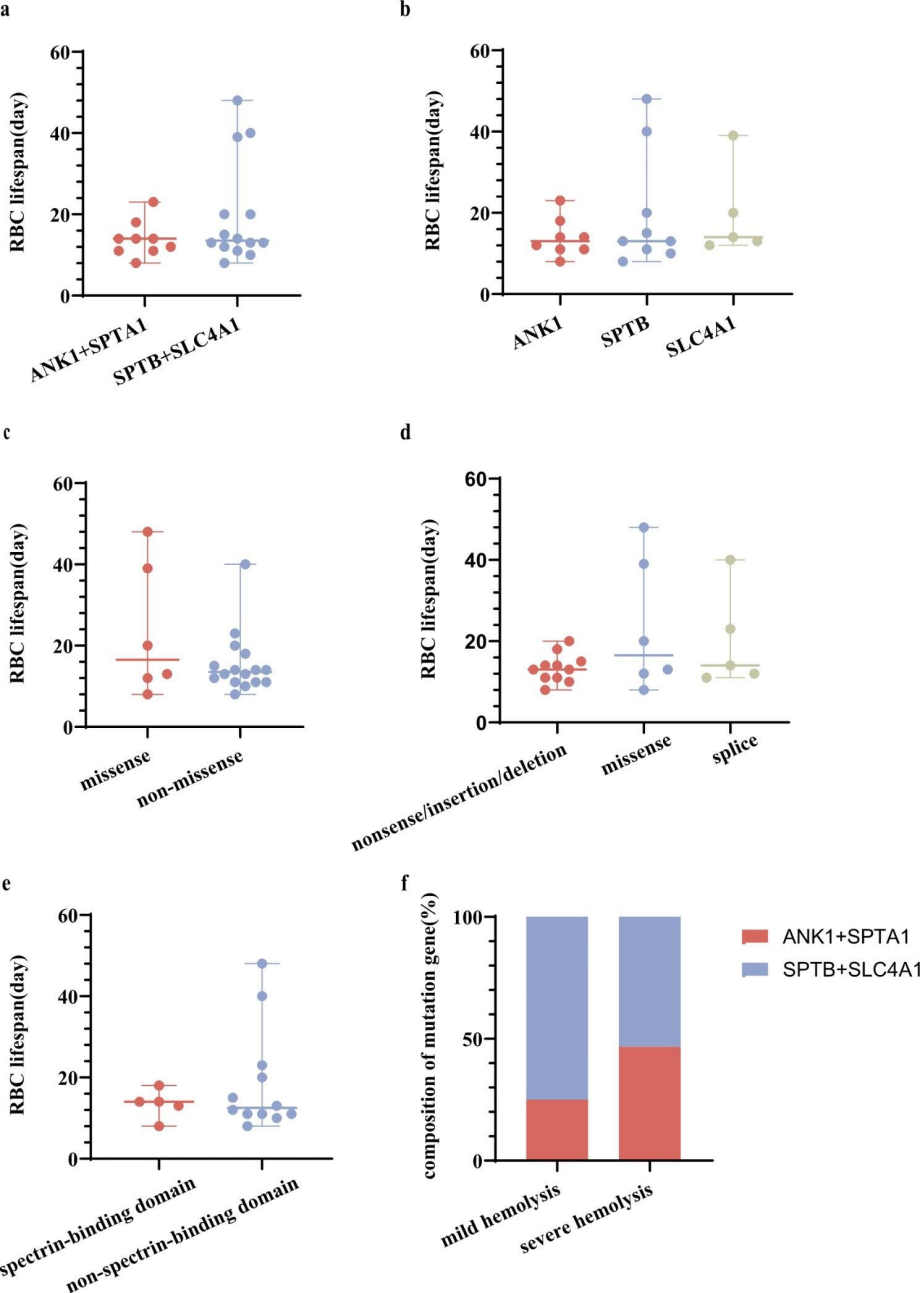
Comparison of RBC lifespan between different mutation genes, types and sites(**a-e**). Mutated gene distribution in different degree of hemolysis (**f**)

The median RBC lifespan was 13 (8–23) days for ANK1, 13 (8–48) days for SPTB and 14 (12–39) days for SLC4A1. Patients with various mutated genes did not exhibit any discernible differences in RBC lifespan (*H* = 0.964, *P* = 0.618). The patient with the SPTA1 mutation had an RBC lifespan of 14 days, which was comparable to that of other patients. The median RBC lifespan in ANK1, SPTA1-HS and SPTB, SLC4A1-HS was 14 (8–23) days and 13.5 (8–48) days, with no statistically significant difference (*U* = 51.500, *P* = 0.477). Notably, 3 patients with RBC lifespans of more than 30 days carried SPTB or SLC4A1 mutations (Fig. 1a-b).

### RBC lifespan in HS with different mutation types

There were 6 missense mutations, 8 insertion/deletion mutations, 3 nonsense mutations and 5 splicing mutations in 22 patients with a single mutation. The median RBC lifespan for missense and nonmissense mutations was 16.5 (8–48) days and 13.5 (8–40) days, respectively, with no significant difference (*U* = 38.500, *P* = 0.494). The median RBC lifespan for missense, splicing and nonsense/insertion/deletion mutations was 16.5 (8–48), 14 (11–40) and 13 (8–20) days, respectively, and no significant difference was found these among groups (*H* = 1.332, *P* = 0.514). Four patients with RBC lifespans exceeding 20 days carried missense or splicing mutations (Fig. 1c-d).

### RBC lifespan in HS with different mutation sites

**Fig. 2:**
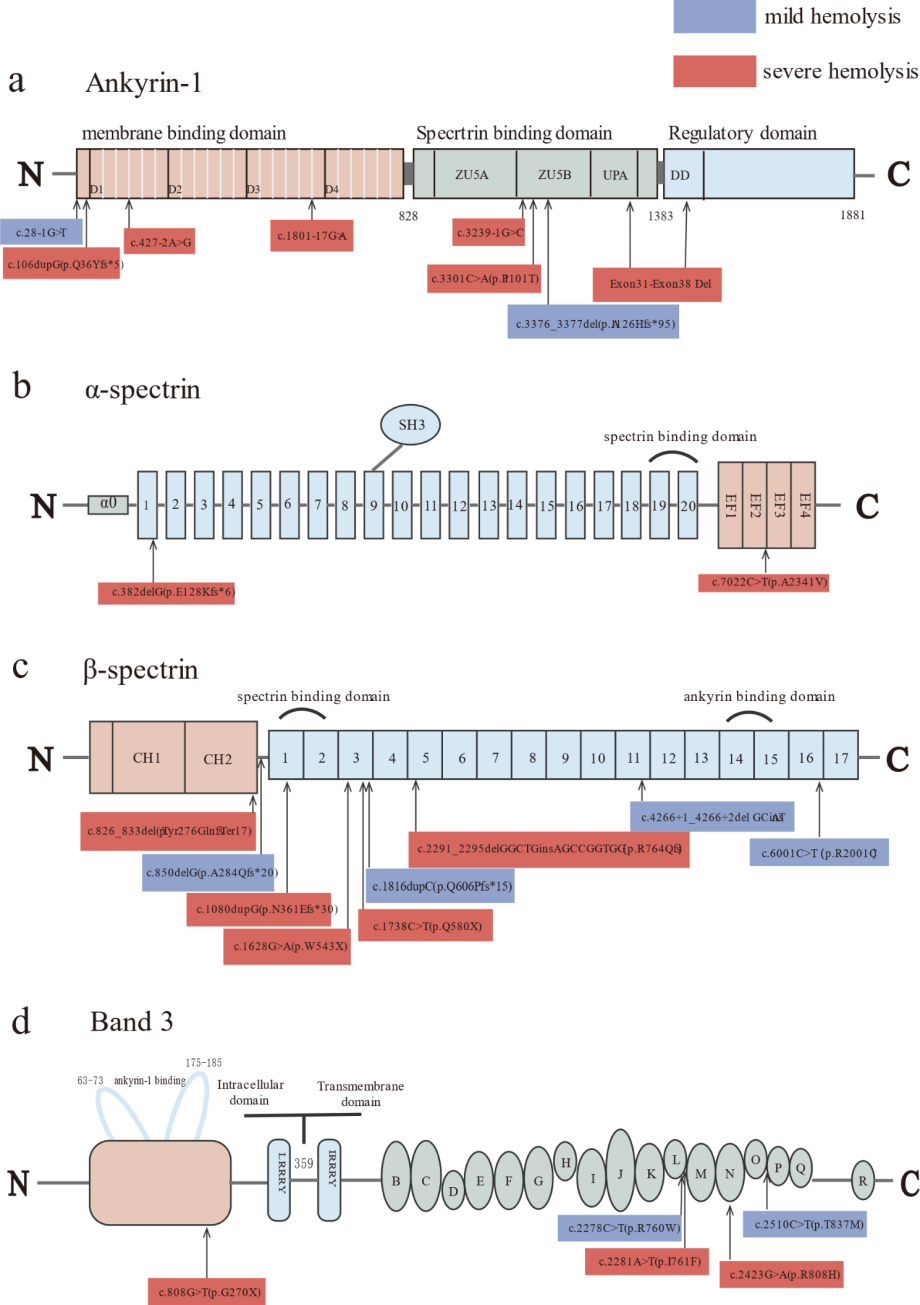
The spectrum of mutations mapped along the protein structures of ankyrin, α/β-spectrin, band 3. Mutations were colored by hemolysis phenotype. The degree of hemolysis was classified as mild(RBC lifespan>14 days) or severe(RBC lifespan ≤ 14 days). Ankyrin [9–12] consists of three domains: a membrane-binding domain, a spectrin-binding domain and a regulatory domain (**a**).α/β-spectrin [13] contains several spectrin-type repeats with specialized domains(**b/c**). Band 3 [14, 15] comprises an intracellular domain and a transmembrane domain (**d**)

In the present study, 22 patients with a single mutation were included. Among the eight ANK1 mutations, four mutations involved the membrane-binding domain, and four mutations involved the spectrin-binding domain. Among the nine SPTB mutations, one mutation involved the junctional region, and eight mutations involved the spectrin repeats. Among the five SLC4A1 variants, one variant involved the intracellular domain, and four variants involved the transmembrane domain (Fig. 2). The 17 patients with non-SLC4A1 mutations were divided into two groups based on their mutation sites, namely, spectrin-binding domain mutations (n = 5) and nonspectrin-binding domain mutations (n = 12); the RBC lifespan of both groups was 14 (8–18) and 12.5 (8–48) days, respectively, with no significant difference (*U* = 29.000, *P* = 0.959) (Fig. 1e). Interestingly, the mutations with RBC lifespans exceeding 20 days were all located in the nonspectrin-binding domain.

### Degree of hemolysis and mutation gene composition

Based on the median RBC lifespan, mild and severe hemolysis were defined as RBC lifespan > 14 days and RBC lifespan ≤ 14 days, respectively. Among the 23 HS patients, there were 8 cases of mild hemolysis, including 2 cases (25%) with ANK1 or SPTA1 mutations and 6 cases (75%) with SPTB or SLC4A1 mutations. In 15 cases of severe hemolysis, 7 patients (46.7%) carried ANK1 or SPTA1 mutations, and 8 patients (53.3%) carried SPTB or SLC4A1 mutations. The composition of the mutated genes did not statistically differ between the two groups (*P* = 0.400) (Fig. 1f).

## Discussion

We analysed the correlation between genotype and degree of hemolysis in 23 HS patients utilizing the RBC lifespan detected by Levitt’s carbon monoxide (CO) breath test. This is the first study to measure RBC lifespan by Levitt’s CO breath test to assess the degree of hemolysis in HS alone, excluding the effect of bone marrow hematopoietic compensation. In the present study, no clear correlation between the genotype and degree of hemolysis was identified.

Clinical studies exploring genotype-phenotype corrections in HS are controversial. Tole et al. [3] reported that children with SLC4A1 mutations have the mildest phenotype, highest Hb levels, lowest reticulocyte counts and lowest unconjugated bilirubin levels. However, van Vuren et al. [4] found that mutations in ANK1 and SPTB generate a more severe clinical phenotype characterized by lower Hb levels and higher reticulocyte counts compared to mutations in SPTA1. Because Hb, as an indicator of the HS phenotype, is affected by both the degree of hemolysis and hematopoietic compensation, it does not objectively reflect the effect of genotype on the clinical phenotype in HS. Therefore, we evaluated RBC lifespan by Levitt’s CO breath test to detect the degree of hemolysis independently, and we found that the degree of hemolysis in HS was unrelated to membrane skeletal protein mutation genes, mutation types or mutation sites.

Ankyrin-1 plays a central role in stabilizing the erythrocyte membrane by attaching spectrin dimers to the transmembrane domain of band 3. Concomitant spectrin and ankyrin-1 deficiency are observed in the erythrocytes of HS patients, and it is postulated that aberrant ankyrin-1 expression may contribute to secondary spectrin deficiency [16]. Previous studies have shown more severe anemia and a higher rate of splenectomy in ANK1-HS than SPTB-HS [5, 6], suggesting that ANK1-HS manifests more severe hemolysis. Tole et al. concluded that children with SPTA1-HS have the most severe clinical phenotype with the lowest Hb levels, and they reported that almost all patients underwent splenectomy in early childhood [3]. In the present study, 3 patients with RBC lifespans of more than 30 days carried SPTB or SLC4A1 mutations, and patients with ANK1 or SPTA1 mutations showed a trend towards more severe hemolysis. However, there was no significant difference in RBC lifespan between different gene mutations, which may have been due to sample size constraints.

The types of mutations in HS can be categorized as missense and nonmissense mutations. Missense mutations that interfere with a single interaction within the ankyrin complex have little effect on the membrane skeleton [10], suggesting that missense mutations may generate a relatively modest hemolytic phenotype. In the present study, there was no significant difference in RBC lifespan between missense and nonmissense mutations. Notably, 2 patients with an RBC lifespan of nearly 40 days both carried missense mutations, suggesting that missense mutations may be associated with milder hemolysis. However, a larger sample size is required to further investigate the association between the degree of hemolysis and mutation types. It has been previously suggested that nonmissense mutations mainly result in the premature introduction of stop codons, leading to either expression of truncated protein or nonexpression of relevant alleles, rather than impacting protein function [10], implying that nonmissense mutations may induce comparable phenotypes. However, the data reported by van Vuren did not support this hypothesis; they proposed that the incorporation of truncated proteins would disrupt cytoskeleton function, suggesting that nonmissense mutations leading to truncated protein expression would result in more severe clinical manifestations compared to allelic deletions that reduce the amount of normally formed protein [4]. In the present cohort, one patient with an SPTB splicing mutation had a considerably longer RBC lifespan compared to other nonmissense mutations. Although the mutation altered the mRNA splicing process, which generated truncated protein expression, the produced protein preserved crucial structural domains, such as the spectrin-binding domain, resulting in a lesser impact on the membrane skeleton and ultimately milder hemolysis. This finding suggested that whether the incorporated truncated proteins preserve essential structural domains may also affect the degree of hemolysis in HS.

The distribution of mutation regions in membrane proteins has been reported to be a potential factor influencing the clinical phenotype. It has previously been demonstrated that the interaction between ankyrin and β-spectrin is critical for erythrocyte deformability and stability. Mutations in the spectrin-binding domain of ANK1 disrupt this interaction, which results in more severe cytoskeleton assembly or function disruption, thereby leading to a more severe clinical phenotype [5]. Similarly, van Vuren et al. [4] proposed that mutations in the ANK1, SPTB and SPTA1 spectrin-binding domains contribute to more severe phenotypes. In the present cohort, four HS patients with mutations in the nonspectrin-binding domain had a longer RBC lifespan, suggesting that mutations in the spectrin-binding domain may lead to a more severe hemolytic phenotype. However, the present study did not demonstrate a clear relationship between the degree of hemolysis and mutation sites, which may be attributed to the small sample size.

Notably, Wang et al. [17] reported that two HS patients with the same genotype show distinct levels of MCV and MCHC, suggesting that the two individuals have different clinical phenotypes. Because the methylation level of the ANK1 promoter region is associated with ANK1 expression [18], we speculated that the varied ANK1 expression, which was due to the variance in methylation levels of the ANK1 promoter region, contributed to the heterogeneity of the hemolytic phenotype in patients with the same genotype. Factors, such as the level of methylation in the promoter region, altered gene expression levels, accounting for the variable degree of hemolysis.

The present study had several limitations. First, because the majority of the patients included in the present cohort were adults, the more severe phenotype of HS as children may have been underreported. Second, certain severe mutations are lethal, and the absence of extremely severe HS cases may result in an inaccurate conclusion. Finally, the small sample size may have skewed the results. With the expansion of the sample size and additional research, more detailed and comprehensive results will be obtained.

## Conclusion

To our knowledge, this is the first study focusing on genotype-hemolytic phenotype association in HS. In the present study, we measured RBC lifespan by Levitt’s CO breath test to assess the degree of hemolysis in HS alone and found no clear correlation between the genotype and degree of hemolysis in HS. Because both hemolysis and bone marrow hematopoietic compensation influenced the severity of clinical manifestations, we hypothesized that the genotype was not the source of the variability of HS clinical presentation.

## Materials and methods

### Patients

We conducted a retrospective study on 23 patients diagnosed with HS at the Blood Diseases Hospital, Chinese Academy of Medical Sciences & Peking Union Medical College. According to the guidelines from the British Society for Hematology [19], diagnosis was based on family history, clinical manifestations, erythrocyte morphology and hematological parameters. All patients had detectable erythrocyte membrane protein gene mutations that were predicted to be pathogenic by next-generation sequencing (NGS), and all patients also had shortened RBC lifespan as determined by Levitt’s carbon monoxide (CO) breath test. None of the 23 patients had been infected within 2 months before enrollment, had hemolytic anemia other than HS or had undergone splenectomy. All patients were from 23 independent family lines.

### Laboratory tests

A fully automated hematology analyser (SysmexXN-9000) was used to measure hematological parameters, including Hb, absolute reticulocyte count (ARC), reticulocyte ratio, mean corpuscular volume (MCV) and mean corpuscular hemoglobin concentration (MCHC). A fully automated chemiluminescence immunoassay (CouterDX-1800) was used to detect erythropoietin and indirect bilirubin concentrations. EMA binding tests were performed according to the literature [20], and the degree of EMA marker deficiency was expressed as a percentage reduction in the mean fluorescence intensity (MCF) of EMA-labelled band 3 protein complexes in HS patients. The osmotic fragility test was performed according to the literature [20], using a biological reference interval (NaCl concentration) of 0.44–0.48% for initiation of hemolysis and 0.28–0.36% for complete hemolysis, and an increase in either of which was considered positive.

### Genetic analysis

Genomic DNA extraction from peripheral blood was performed using a DNA extraction kits (Beijing Tiangen Biochemical Technology Co., Ltd.) and QIAamp DNA Mini Kit (Qiagen, Germany). The targeted NGS panel included the coding exons, and some meaningful intronic regions of the genes known to be associated with the RBC membrane, enzyme disorders, congenital dyserythropoietic anemia, hemoglobinopathies and bilirubin metabolism. DNA libraries were built using the GenCap Custom Kit (MyGenostics, Beijing) targeting the mentioned genes (ANK1, SPTB, SLC4A1, SPTA1 and EPB42). Biotin-labelled capture probes (80-120-mer) were used to cover all exons with nonrepeat regions. The average gene coverage of the targeted regions was 94.86%, and the average sequencing depth was 436.75×. In addition, 90.14% of the targeted regions covered > 30×, and 60.29% of the targeted regions covered > 200×. Genetic variations were compared against the Human Gene Mutation Database (https://www.hgmd.cf.ac.uk/ac/index.php), ClinVar (http://www.ncbi.nlm.nih.gov/clinvar) and dbSNP database (https://www.ncbi.nlm.nih.gov/snp). Mutation pathogenicity was predicted using Protein Variation Effect Analyser (PROVEAN, http://provean.jcvi.org) and MutationTaster(https://www.mutationtaster.org). Pathogenicity was evaluated according to the guidelines of the American College of Medical Genetics and Genomics [8].

### RBC lifespan analysis

Levitt’s CO breath test, which has been demonstrated to be not inferior to the [15] N glycine labelling technique [21], was applied to detect RBC lifespan. The RBC lifespan was measured as the total capacity of CO from Hb degradation divided by the quantity of CO released per day. The ELS Tester (Seekya Biotec Co. Ltd. Shenzhen, China) was used to determine endogenous CO concentration by nondispersive infrared spectroscopy with air-alveolar gas-paired samples. After the power was connected, the alveolar and environmental samples were connected to inlets, and the given Hb concentration of the subject for that day was entered. The measurement mode was turned on, triggering the instrument to start a series of automated measurements, which were completed within 15 min.

Blood samples, alveolar gas, and environmental background air were collected for the test. Blood samples were collected using EDTA anticoagulation tubes to determine Hb concentration. Alveolar air samples were collected in the morning (8:00 a.m. − 11:30 p.m.) 10 min before or after blood sampling. After a deep inhalation, each subject was instructed to hold his or her breath for 10 s and then exhale into the collection system through a mouthpiece. The system discarded the first 300 ml of volume, which was considered to contain dead space gas, and it then directed subsequent alveolar air into a foil collection bag. If needed, the procedure was repeated until the collected air sample reached 1000 ml. The 1000-ml bag was detached and sealed immediately. Atmospheric samples were collected simultaneously with alveolar gas collection. Air samples were stored at room temperature and analysed within 5 days.

### Statistical analysis

SPSS 26.0 software was applied for statistical analysis. Continuous variables are described as medians (ranges), and the Kruskal–Wallis *H* test or Mann–Whitney *U* test was used for comparisons between groups. The significance of differences in categorical variables between groups was determined using Fisher’s exact test. All tests were two-tailed, and a *p value* of < 0.05 was considered statistically significant.

## Data Availability

The data supporting this study’s findings are available from the corresponding author upon reasonable request.

## List of Abbreviations

HS: Hereditary spherocytosis
RBC: Red blood cell
NGS: Next-generation sequencing
CO: Carbon monoxide
Hb: Hemoglobin
IBIL: Indirect bilirubin
MCV: Mean corpuscular volume
MCHC: Mean corpuscular hemoglobin concentration
ARC: Absolute reticulocyte count
Ret: Reticulocyte
EPO: Erythropoietin
